# The Australian Ovarian Cancer Study

**DOI:** 10.1186/1897-4287-10-S2-A94

**Published:** 2012-04-12

**Authors:** N Traficante, S Fereday, L Galletta, J Hung, D Giles, K Alsop, J Hendley, A Iuga, G Chenevix-Trench, A Green, P Webb, A DeFazio, D Bowtell

**Affiliations:** 1 Peter MacCallum Cancer Centre, East Melbourne, VIC, Australia; 2 Department of Gynaecological Oncology, University of Sydney at Westmead Millennium Institute, Westmead Hospital, Westmead, NSW, Australia; 3 Westmead Institute for Cancer Research, University of Sydney at Westmead Millennium Institute, Westmead Hospital, Westmead, NSW, Australia; 4 Queensland Institute of Medical Research, Brisbane, Qld, Australia; 5 Department of Biochemistry and Molecular Biology, University of Melbourne, Parkville, VIC, Australia

## Background

AOCS commenced in 2000 as a collaborative study between researchers at the Peter MacCallum Cancer Centre (PMCC), Queensland Institute for Medical Research (QIMR), Westmead Institute for Cancer Research (WICR) and University of Melbourne. Patient recruitment ceased on June 30, 2006. AOCS recruited a total of 1834 women with invasive or borderline ovarian cancer, far exceeding the initial target. We have received a total of 1815 completed questionnaires and have collected 1080 fresh tumour tissue samples and 1582 blood samples. Control recruitment is also complete and a total of 1066 control women that did not have ovarian cancer were recruited. Clinical details have been recorded for all AOCS patients, with clinical follow-up done at 6-monthly intervals: we have primary treatment data, including surgery and chemotherapy details on 99% of cases; and 89% of eligible cases have follow-up to five years post-diagnosis.. Thus far only 123 patients (6.5%) have been lost to follow-up, despite that fact that 30-40% of our patients return to regional areas for ongoing treatment.

## Relapse disease and collection of ascites

We are collecting ascites and tumour tissue (excess to diagnostic requirements) from women who present with recurrent disease. To date we have collected 337 ascitic fluid samples from 214 patients, with scientific analysis of these cases currently underway.

## AOCS resource

AOCS is a resource for both local and international researchers who can apply to access biospecimens and associated data. Materials available include DNA (tumour and germline), RNA, plasma, serum, fresh frozen tissue, formalin-fixed paraffin embedded (FFPE) blocks, Tissue Microarrays (TMA) and extensive clinical and epidemiological data. To date we have approved approximately 70 national and international projects and collaborations, some of which are listed below-

• SWI/SNF mutations in clear cell carcinoma (BC Cancer Agency, Vancouver, Canada)

• International Cancer Genomics Consortium Study (PMCC, Melbourne, Aus)

• HER2 overexpression and amplification is present in a subset of ovarian mucinous carcinomas and can be targeted with trastuzumab therapy (University of British Columbia, Vancouver, Canada)

• Genetic relationships between breast and ovarian cancer: shared chromosomal alterations and response to chemotherapy (Dana Faber Cancer Institute, Boston, USA)

• Quality of life among women with platinum-resistant and platinum-sensitive ovarian cancer who were treated for recurrence: A prospective study (QIMR, Brisbane, Aus)

• Development of a novel xenograft mouse model of high grade epithelial ovarian cancer for analysis of therapeutic manipulation in order to improve outcomes for patients with ovarian cancer (Walter and Eliza Hall Institute, Melbourne, Aus)

• Deep sequencing of 19p13 (National Cancer Institute, Maryland, USA)

Application forms and a complete list of approved projects can be found at http://www.aocstudy.org.

